# Advancing solar energy conversion materials: fuel the future

**DOI:** 10.1093/nsr/nwab128

**Published:** 2021-07-15

**Authors:** Zhigang Zou

**Affiliations:** Eco-Materials and Renewable Energy Research Center (ERERC), Jiangsu Key Laboratory for Nano Technology, National Laboratory of Solid State Microstructures, School of Physics, Nanjing University, China

In the fight against climate change, achieving carbon neutrality has become a severe test and task for global society. Meanwhile, this is also a great opportunity for decarbonization, mitigating climate risks and supporting the transition to a renewable energy future. Hopefully, a variety of future-oriented ‘black technologies’ will emerge and grow immediately. In particular, the birth and development of solar energy conversion products has been entering contemporary society, and has become an indispensable key link of low-carbon cycle and green development. Enormous effort has been dedicated to building a comprehensive sustainable system based on solar energy conversion, and it has been a consensus that developing advanced materials is the basis of realizing high-efficiency, low-cost solar energy conversion and utilization.

In recent years, photovoltaic and artificial photosynthetic systems with traditional materials and structures have become increasingly mature, and solar energy conversion systems represented by silicon-based semiconductors have been widely used all over the world. However, due to the serious pollution caused by crystalline silicon modules in the production process, and the relatively low theoretical conversion efficiency of silicon-based semiconductors, a variety of new photovoltaic materials and devices based on novel materials have been proposed. In particular, organic and perovskite solar cells have attracted wide attention. In addition, since the responsive range of the solar spectrum spread from ultraviolet to the visible region at the beginning of the 21st century, solar photosynthetic processes have obtained extensive attention in the fields of physics, chemistry, materials and energy. Photosynthesis has become an important branch of catalytic chemistry and energy materials because of the continuous development of the catalytic theory, the increasing number of material systems, the improvement of quantum efficiency and the expanding of the application range. All this progress expedites the delivery of this special issue.

On this topic, some outstanding and dedicated scientists provided their views on advanced materials for photovoltaics and artificial photosynthesis, especially the opportunities and challenges of the future. I believe this will inspire the development of solar energy conversion both at home and abroad. The Perspective article from Professor Yingping Zou on the research progress and future direction of the electron acceptor Y6 and its derivatives provides important guidelines for future research into high-performance organic solar cells. Professor Jinglong Gong, Professors Fujun Zhang and Han Young Woo, and Professors Jianhui Hou and Shaoqing Zhang introduced their material research on organic solar cells and photocatalytic H_2_ production, respectively, expanding the potential application of state-of-the-art solar energy conversion devices. Professor Zhiyong Jason Ren, Professors Ding-Jiang Xue, Yuan Lin and Jing-Song Hu, Professors Xiao Wang and Jiangyu Li, Professors Zhiliang Ku, Jianfeng Lu and Fuzhi Huang, Professor Junbai Li, and the professors of my research team systematically reviewed and highlighted the recent progress made with perovskite solar cells and artificial photosynthesis, ranging from fundamental science to application. All the above together presented an overview of solar energy conversion materials research in China.

I would like to thank the members of the editorial office of NSR  for their diligent work. In addition, without the support and help from invited experts and contributors, I would not be able to complete my guest editing work. I hope that all the papers in this special topic will be beneficial to the readers, from general readers and new learners such as graduate students, to experts in this field.

